# Vedolizumab (Entyvio®) for the Treatment of Pyoderma Gangrenosum in a Crohn’s Disease Patient

**DOI:** 10.7759/cureus.12582

**Published:** 2021-01-08

**Authors:** Kevin Groudan, Kamesh Gupta, Rohit Singhania

**Affiliations:** 1 Internal Medicine, Baystate Medical Center, Springfield, USA; 2 Gastroenterology, Baystate Medical Center, Springfield, USA

**Keywords:** inflammatory bowel disease, pyoderma gangrenosum, vedolizumab, entyvio, crohn's disease, ulcerative colitis

## Abstract

Vedolizumab is a humanized monoclonal integrin blocker with gut selective effects on lymphocyte trafficking. Its efficacy and safety for the treatment of moderate to severe Crohn’s disease and ulcerative colitis were demonstrated by phase III GEMINI studies (GEMINI 1 trial: Vedolizumab as Induction and Maintenance Therapy for Ulcerative Colitis; GEMINI 2 trial: Vedolizumab as Induction and Maintenance Therapy for Crohn's Disease). Post hoc analyses of the GEMINI studies further showed the potential benefit of vedolizumab for treating various extraintestinal manifestations, including arthralgias, pyoderma gangrenosum, erythema nodosum, and uveitis. However, findings lacked statistical significance highlighting the need for more clinical data describing vedolizumab’s effects on extraintestinal manifestations. There are currently few case reports describing the effect of vedolizumab on pyoderma gangrenosum specifically. We report a Crohn’s disease patient whose severe pyoderma gangrenosum of her legs, abdomen, and face have been inactive since starting vedolizumab.

## Introduction

Vedolizumab (VDZ), sold under the brand name Entyvio®, is a novel monoclonal antibody approved for the treatment of moderate to severe ulcerative colitis (UC) and Crohn’s disease (CD) [1]. Post hoc analyses of the GEMINI trials (GEMINI 1 trial: Vedolizumab as Induction and Maintenance Therapy for Ulcerative Colitis; GEMINI 2 trial: Vedolizumab as Induction and Maintenance Therapy for Crohn's Disease) demonstrated its potential role in treating extraintestinal manifestations like pyoderma gangrenosum (PG) [2-4]. PG is a relatively common extra-intestinal manifestation that contributes significantly to the burden of illness in inflammatory bowel disease (IBD) patients. There are currently few reports of PG treated with VDZ. We report a CD patient whose severe PG of her legs, face, and abdomen have been in remission since starting VDZ.

## Case presentation

A 38-year-old Caucasian woman with complex fistulizing CD, status post subtotal colectomy with ileostomy and complicated by peristomal, facial, and leg PG, presented to IBD clinic with worsening abdominal cramping and increased ostomy output. Her CD had been difficult to manage since her diagnosis at age 30. She was treated before with infliximab, adalimumab, sulfasalazine, and mesalamine, which did not improve her gastrointestinal symptoms or PG. She was steroid dependent for nearly the entire duration of her disease and was unable to taper below 20 mg prednisone daily before her rectal symptoms would flare and skin lesions would “open up.”

**Figure 1:**
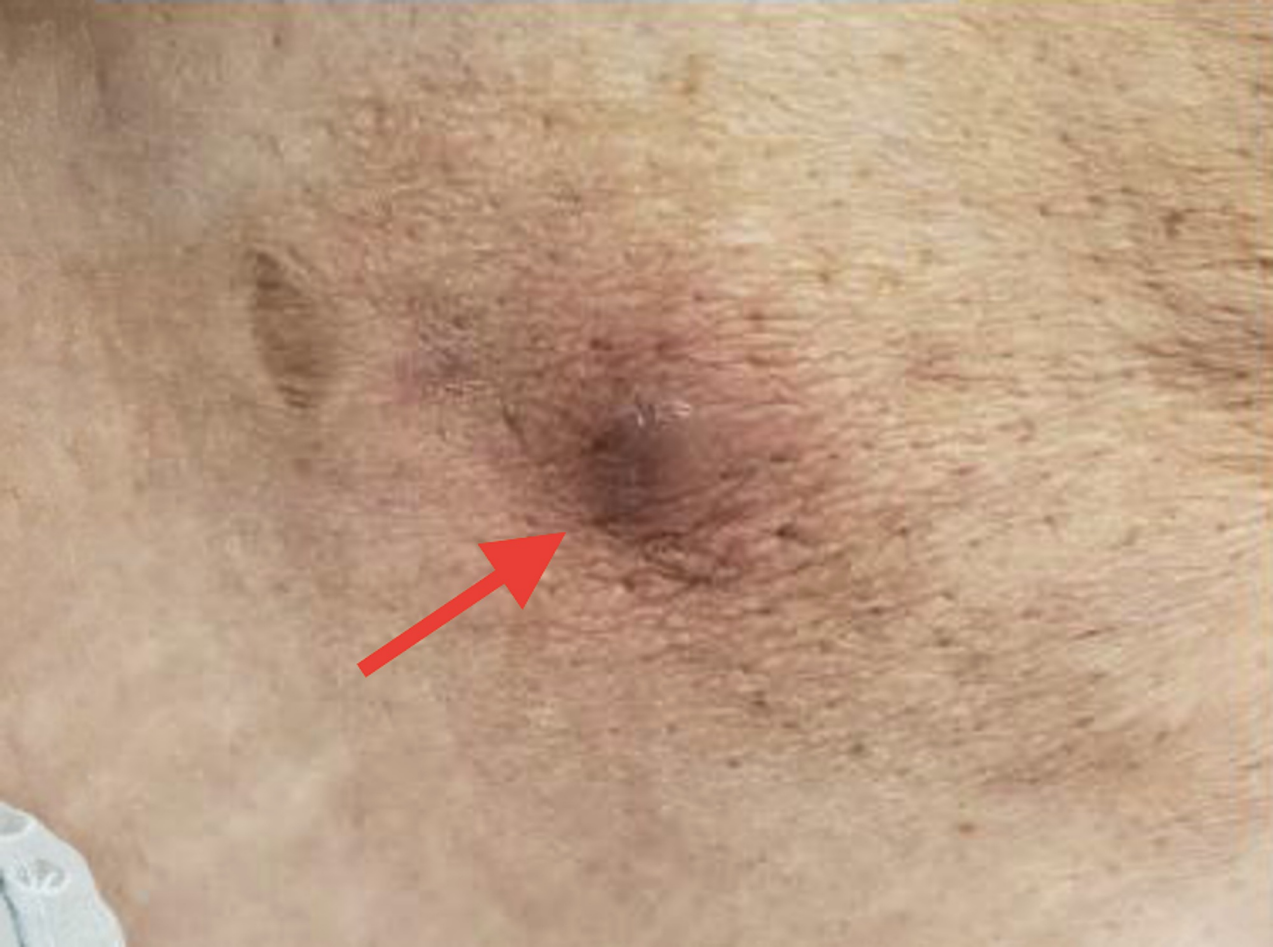
Healed leg pyoderma gangrenosum after taking vedolizumab for approximately one year

**Figure 2:**
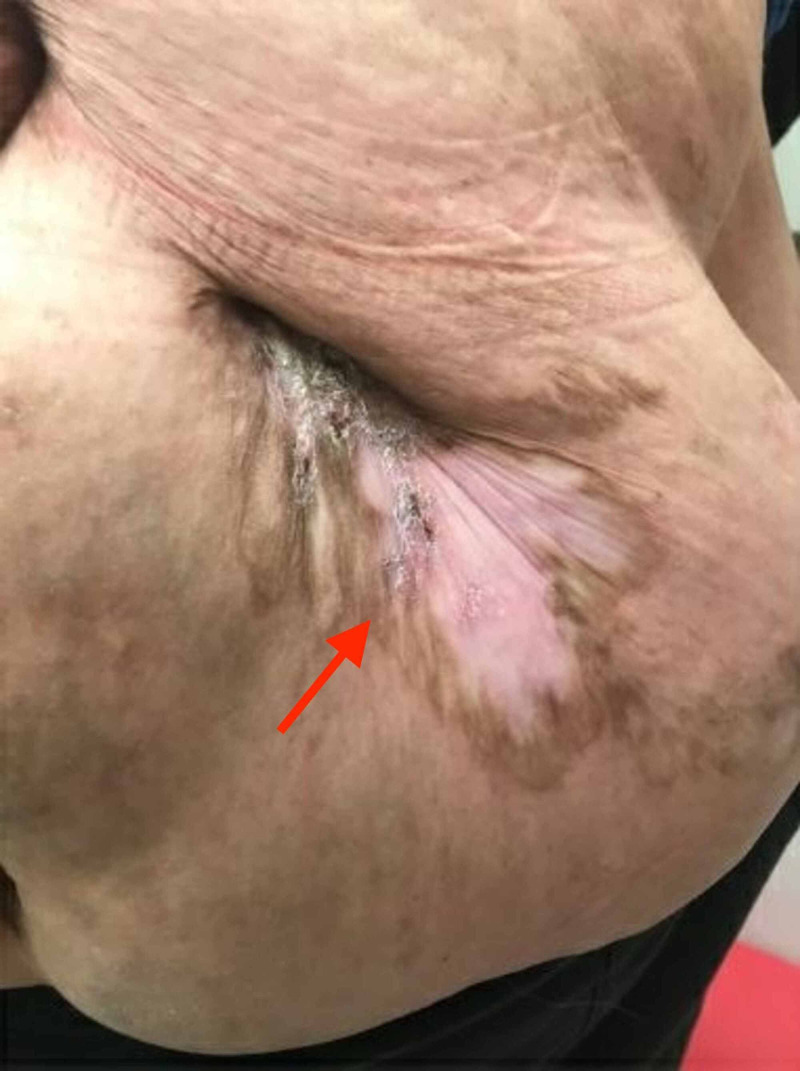
Healed abdominal pyoderma gangrenosum after taking vedolizumab for approximately one year

On arrival, she was vitally stable. Her most recent labwork was significant for an erythrocyte sedimentation rate of 44 (normal: 0-22), C-reactive protein of 8.48 (average: 1.0-3.0), and fecal calprotectin of <15.6 (normal: <50.0). She was started on VDZ induction therapy: 300 mg IV at weeks 0, 2, 6, and 14. After two doses, her leg PG was noted to be nearly healed. Additionally, her abdominal cramping and ostomy output improved, and she was able to taper from 40 mg daily prednisone to 20 mg. After her fourth induction dose, her peristomal, facial, and leg PG were all inactive. She was started on VDZ maintenance infusions: 300 mg IV every eight weeks. At her three months follow-up, she was down to 5 mg of prednisone daily. Healed leg and abdominal pyoderma gangrenosum after taking vedolizumab for approximately one year are presented in Figures 1-2. Three years later, her PG remains inactive on VDZ maintenance infusions and off chronic prednisone.

## Discussion

PG is a non-infectious neutrophilic dermatosis that affects approximately 5% to 12% of UC patients and 1% to 2% of CD patients [5]. PG most commonly presents as an inflammatory papule or pustule that grows into tender ulceration with violaceous borders and a purulent base. Its pathogenesis is not well understood but is thought to involve neutrophil dysfunction, inflammatory dysregulation, and genetic mutations [5]. Aside from IBD, PG is associated with other autoimmune diseases, including rheumatoid arthritis as well as hematologic disease and malignancy [6]. PG is often diagnosed clinically; biopsy can exclude other causes of ulceration. Proposed major diagnostic criteria include identifying "a rapidly progressive painful, necrolytic cutaneous ulcer with irregular, violaceous borders" and exclusion of alternative causes of ulceration. Minor criteria include a history of pathergy/cribriform scarring, history of systemic disease associated with PG, histopathologic features of dermal neutrophilia, mixed inflammation or lymphocytic vasculitis, and rapid response to systemic corticosteroids. The presence of both major criteria and two out of four minor criteria confirm a diagnosis of PG [5].

PG severity is often independent of gastrointestinal IBD disease activity [7]. Clinical features depend on the type of PG. Variants include vesicular-bullous, pustular, ulcerative, and superficial granulomatous. Vesicular-bullous PG typically appears on the upper extremities and face and presents with associated fever and joint pain. Pustular PG is characterized by numerous small pustules that either resolve or progress into painful ulcerations. One of the most characteristic features of PG is its exaggerated response to minor skin injury, a phenomenon known as pathergy. Insults to the skin that are normally benign could be devastating for PG patients. This can complicate decision making to perform certain surgeries and procedures, particularly important in advanced IBD patients who require colectomy [5].

Guidelines recommend corticosteroids as first-line treatment for PG. Limited and mild disease can be treated with intralesional corticosteroids (triamcinolone) [8]. Topical tacrolimus, nicotine, topical dapsone, and sodium cromoglycate have also been used for mild disease. Rapidly growing PG should be treated with systemic corticosteroids or oral cyclosporine [5]. For refractory disease, tumor necrosis factor (TNF) antagonists such as etanercept and adalimumab, the interleukin (IL)-12/23 inhibitor ustekinumab, the IL-1 beta monoclonal antibody canakinumab, and steroid-sparing immunosuppressives like azathioprine have been reported to be effective [8]. Despite adequate treatment, PG can take a long time to heal, remain very painful, and recur. Many patients require opioids for severe pain. A wound care specialist is often needed to educate the patient on preventing wound breakdown and infection [5].

VDZ is a humanized monoclonal antibody that targets the a4B7 integrin receptor of gut-homing T-lymphocytes, inhibiting their translocation from the blood into the inflamed gut [1]. VDZ is unique among conventional biologics because of its gut-selective mechanism of action. VDZ has demonstrated efficacy and safety for treating moderate to severe UC and CD in adult patients based on the phase 3 GEMINI 1 [2] and GEMINI 2 [3] trials, respectively. Although these trials were not designed to assess extra-intestinal manifestations, post hoc analyses of them showed the potential benefit of VDZ in treating extra-intestinal complications such as arthralgia, uveitis, erythema nodosum, and PG [4]. However, their results lacked statistical significance, highlighting the need to further study VDZ’s relationship with extraintestinal manifestations.

VDZ’s role in treating PG specifically remains controversial according to the limited available data. Kodoru et al. reported a 22-year-old CD patient whose leg nearly healed after her fourth dose of VDZ [9]. Fleisher et al. reported an 87-year-old CD patient whose peristomal PG improved with VDZ. They also reported a 24-year-old UC patient whose peristomal PG completely healed after six doses of VDZ [10]. In contrast, Yeh and Tsiaras reported a 32-year-old CD patient who developed multiple PG ulcers on her shins after one month of VDZ [11]. Kim et al. reported a 43-year-old UC patient who developed severe PG of her left breast after starting VDZ [12].

## Conclusions

In conclusion, we present a CD patient whose severe leg, face, and abdominal PG is in remission after starting VDZ therapy. Although VDZ has proven benefit for treating moderate to severe UC and CD, its role in treating extraintestinal manifestations remains unclear. As PG is relatively common and contributes significantly to the IBD disease burden, and given the limited available data, larger cohort studies are needed to elucidate the role of VDZ in treating PG.
